# Using routinely available electronic health record data elements to develop and validate a digital divide risk score

**DOI:** 10.1093/jamiaopen/ooaf004

**Published:** 2025-02-04

**Authors:** Jamie M Faro, Emily Obermiller, Corey Obermiller, Katy E Trinkley, Garth Wright, Rajani S Sadasivam, Kristie L Foley, Sarah L Cutrona, Thomas K Houston

**Affiliations:** Department of Population and Quantitative Health Sciences, University of Massachusetts Chan Medical School, Worcester, MA 01605, United States; Department of Internal Medicine, Wake Forest University School of Medicine, Winston-Salem, NC 27101, United States; Department of Internal Medicine, Wake Forest University School of Medicine, Winston-Salem, NC 27101, United States; Department of Family Medicine, University of Colorado School of Medicine, Aurora, CO 80045, United States; Department of Clinical Pharmacy, University of Colorado School of Medicine, Aurora, CO 80045, United States; Department of Population and Quantitative Health Sciences, University of Massachusetts Chan Medical School, Worcester, MA 01605, United States; Department of Implementation Science, Wake Forest University School of Medicine, Winston-Salem, NC 27101, United States; Department of Population and Quantitative Health Sciences, University of Massachusetts Chan Medical School, Worcester, MA 01605, United States; Center for Health Optimization and Implementation Research, Veterans Affairs Bedford Healthcenter System, Bedford, MA 01730, United States; Department of Internal Medicine, Wake Forest University School of Medicine, Winston-Salem, NC 27101, United States

**Keywords:** digital divide, health literacy, electronic health record, health care access, screening tool

## Abstract

**Background:**

Digital health (patient portals, remote monitoring devices, video visits) is a routine part of health care, though the digital divide may affect access.

**Objectives:**

To test and validate an electronic health record (EHR) screening tool to identify patients at risk of the digital divide.

**Materials and Methods:**

We conducted a retrospective EHR data extraction and cross-sectional survey of participants within 1 health care system. We identified 4 potential digital divide markers from the EHR: (1) mobile phone number, (2) email address, (3) active patient portal, and (4) >2 patient portal logins in the last year. We mailed surveys to patients at higher risk (missing all 4 markers), intermediate risk (missing 1-3 markers), or lower risk (missing no markers). Combining EHR and survey data, we summarized the markers into risk scores and evaluated its association with patients’ report of lack of Internet access. Then, we assessed the association of EHR markers and eHealth Literacy Scale survey outcomes.

**Results:**

A total of 249 patients (39.4%) completed the survey (53%>65 years, 51% female, 50% minority race, 55% rural/small town residents, 46% private insurance, 45% Medicare). Individually, the 4 EHR markers had high sensitivity (range 81%-95%) and specificity (range 65%-79%) compared with survey responses. The EHR marker-based score (high risk, intermediate risk, low risk) predicted absence of Internet access (receiver operator characteristics *c*-statistic=0.77). Mean digital health literacy scores significantly decreased as her marker digital divide risk increased (*P*  <.001).

**Discussion:**

Each of the four EHR markers (Cell phone, email address, patient portal active, and patient portal actively used) compared with self-report yielded high levels of sensitivity, specificity, and overall accuracy.

**Conclusion:**

Using these markers, health care systems could target interventions and implementation strategies to support equitable patient access to digital health.

## Background

Implementation of digital health strategies in clinical settings has increased. Examples of these strategies include video telehealth visits, text-message reminders for upcoming appointments, health-related apps, and patient portals where patients can access their health records, request appointments and medication refills, and send secure electronic messages to their clinical teams.^1^ The COVID-19 pandemic has further sped up the implementation of these tools, as many health care systems were forced to deliver their care virtually.^2^ Digital health may improve effectiveness of health care and reduce health care barriers. However, if digital health is increasingly considered part of health care access but not all patients have equal access to digital health, health care disparities might be exacerbated.^3–6^

Technology access inequality has been described as the digital divide.^7^ Increasing access to technology and the Internet was proposed as a social determinant of health in Healthy People 2010 and subsequent reports. Although access has improved, with 81% of US adults now owning a smartphone and 75% owning a laptop or desktop computer,^8^ gaps remain for lower income and rural families. Further, access to technology is just a first step in optimally using digital health tools. The Digital Health Equity framework integrates the digital determinants of health and the social determinants of health and describes how these factors are interconnected to worsen the digital divide.^9^ Digital determinants include factors such as technology access, technology literacy, beliefs, values, and cultural norms. If health care systems are to develop programs to support patients and families in overcoming barriers to digital health tools, patients at risk for the digital divide need to be identified. As health care systems are implementing new screening questions for social determinants of health (eg, housing, food, transportation insecurity), they could also add assessments to screen for technology access inequality.^7^ However, implementation of new patient questionnaires can be challenging.

Consequently, we explored the feasibility of using data elements that are already routinely available in electronic health records (EHRs) as a pragmatic assessment of the “digital divide.” If the data elements (eg, documentation of patient cellular phone number, email address, registration with patient portal, and use of the patient portal) were available for most patients in most EHRs, they could be used to identify patients’ digital divide risk. In this report, we describe an initial validation of individual data elements and their combination into an EHR digital divide risk score. We tested the validity of the risk score against patient self-report of lack of Internet access. Secondarily, we evaluated whether the risk score predicted patient self-report of lower technology use and technology literacy.

## Methods

### Study design

Within a health care system integrated across a multistate region in the southern United States, we used the EHR to identify a retrospective cohort of patients. These patients were then asked to complete a cross-sectional survey related to technology and health. Survey results were then combined with the EHR data. Below we describe (1) the approach to identifying data elements as potential markers of the digital divide, (2) the survey content, (3) the patient sample and survey implementation, and (4) statistical analyses. All aspects of the study, including EHR data and survey elements were approved by an Institutional Review Board.

### Digital divide marker identification

We conducted meetings with health care professionals (physicians and nurses), medical coders, and health information technology professionals across 3 US health care systems (1 southern, 1 northeastern, and 1 western) participating in an NIH-funded implementation science consortium.^10^^,^^11^ We asked these key informants to suggest EHR data elements (those routinely collected in the clinical systems) that would potentially be associated with the digital divide. We then further refined our EHR markers of interest using specific criteria including that the data element (1) had preliminary face validity related to technology access we could confirm in our analysis; (2) was extractable as a discrete field; (3) was noted as commonly available across multiple, separate EHR systems; and (4) was routinely collected on most or all patients across primary care and subspecialty settings (ie, less than 80% complete were considered low priority).

Using these criteria, 4 data elements were selected for validation as digital divide markers. First, does the patient have a cellular phone listed in the EHR (those with only a home phone number and no mobile phone were considered at risk)? Second, does the electronic record include the patient’s email address? The presence of these 2 data elements relate to general technology access and use.

The next 2 data elements relate to the patient interacting with the patient portal connected to the EHR. Does the patient have a patient portal account? If a patient registered for the portal and has an active portal account, this suggests they may be less likely to be at risk of the digital divide. Finally, to measure a patient’s engagement with their patient portal, we extracted the number of patient portal logins in the past year. Patients with 2 or more logins in the past year were considered actively using the portal.

### Survey content

In the patient survey, each EHR data element had a corresponding question. Patients were also asked about general access to the Internet (by computer or cellular phone). Additional questions about general technology use, use of technology for health or health care reasons, digital health literacy, and general health literacy. Patient characteristics including demographics, socioeconomics, and social determinants were also included. We used previously validated items from the Health Information National Trends Survey,^12^ the Behavioral Risk Factor Surveillance System,^13^ and the PEW Research Center.^14^ To measure eHealth literacy, we used the full version of eHEALS: The eHealth Literacy Scale, an 8-item measure of eHealth literacy.^15^

### Study sample

Survey participants were selected from a cohort of adult patients (18 years and older) with at least 1 clinic visit in the prior 12 months (*n* = 303 896). We used a purposeful sampling strategy to assure inclusion of racial and ethnic minorities, older adults, and those from rural areas. To assure a range of EHR markers, the sample was stratified by (1) patients with all 4 EHR data elements suggesting low digital divide risk (cell phone documented, email documented, patient portal active, and patient using patient portal), (2) patients with all 4 suggesting high risk (no cell phone, no email, portal not active, and not using portal), and (3) intermediate risk, patients with some data elements positive (1-3 risk factors).

Surveys were mailed with a $1 cash incentive, and an additional $50 gift card was sent to those returning completed surveys, a successful method used in prior trials.^16^ To increase the response rate, each participant who did not respond within 1 month of mailing the survey received a follow-up post card and 2 weeks later were sent another survey packet.

### Statistical analysis

Statistical analyses were conducted to (1) compare each EHR data element with its corresponding survey item, (2) measure the association of the combined EHR data elements’ digital divide risk score with patient report of Internet access, and secondarily (3) measure the association of the digital divide risk score and survey measures related to the digital divide (including technology use and eHealth literacy). All analyses were performed using R statistical software, version 4.2.3.

#### Validation of individual EHR data elements

Using patient survey report as the standard, we constructed a 2×2 contingency table. We calculated the sensitivity, specificity, and overall accuracy of each EHR digital divide marker. For this primary analysis, we approached missing survey data in the following way. To assess the impact of missingness, we conducted a sensitivity analysis where we first assigned all missing values for each survey item to a positive response (eg, access to patient portal) and then conducting the analyses with each survey item assigned to a negative response. Results were then compared with the primary, complete case, analyses to see whether the missing data results were within the 95% CIs of the primary analyses.

#### Predicting lack of Internet access with EHR digital divide risk score

The 4 EHR digital divide markers were combined to represent higher risk, intermediate risk, and low risk. For this analysis, the dependent variable was patient-reported lack of access to the Internet and independent variable was the digital divide risk score. The probability of lack of Internet was calculated for each level of digital divide risk using logistic regression and odds ratios were calculated. From the model, a receiver operator characteristics (ROC) curve was generated and area under the ROC curve (*c*-statistic) was calculated.

#### Association with additional digital divide factors

We assessed the association of the EHR digital divide risk score with general technology use, use of technology for health or health care reasons, and digital health literacy (eHEALS). Significance in trends was observed in survey responses across the ordinal levels of digital divide risk (low, intermediate, high) by using the Cochran Armitage test for categorical outcomes and the Jonckheere-Terpstra test for quantitative outcomes. In addition, we evaluated the association of patient characteristics with the risk score.

## Results

We distributed surveys to 632 individuals, and 249 people completed the survey (response rate = 39.4%). Those who responded were not meaningfully different (less than 9%) in terms of age distribution, sex, and income. However, there were larger differences in response rates based on race/ethnicity. A higher proportion identifying as “White or Caucasian” responded in comparison to those identifying as “Black or African American” and “Hispanic/Latino” (see Table S2). Overall, our purposeful sampling strategy succeeded in recruiting a diverse sample in terms of demographic, geographic location, and social determinants (Table 1). Among respondents, approximately half (53.4%) were aged 65 and older, and 50.2% were non-White (23.3% were Black or African American and 26.9% other race) with 10.6% Hispanic, with the sample included those living in rural areas (24.5%) and small towns (30.9%), based on RUCA 2010 codes. Across the additional social determinant questions, about 20% reported income, housing, or food insecurity concerns.

**Table 1.** Characteristics of participants (*n*=249) responding to digital divide survey.

		Respondents
	Variable	*n* =249
Demographic characteristics	Age	
	18-34	32 (12.9%)
	35-54	34 (13.7%)
	55-64	50 (20.1%)
	65+	133 (53.4%)
	Gender	
	Female	128 (51.4%)
	Male	121 (48.6%)
	Race	
	White	124 (49.8%)
	Black	58 (23.3%)
	Other race	67 (26.9%)
	Ethnicity	
	Hispanic/Latino	26 (10.4%)
	Not Hispanic/Latino	211 (84.7%)
	Missing	12 (4.8%)
Urban-rural location	Metropolitan or Micropolitan	111 (44.6%)
	Small town	77 (30.9%)
	Rural	61 (24.5%)
Health insurance	Commercial	116 (46.6%)
	Medicaid	20 (8.0%)
	Medicare	113 (45.4%)
Educational status	Did not graduate high school	28 (11.2%)
	Finished high school or GED	77 (30.9%)
	Some college	47 (18.9%)
	Associate’s degree	21 (8.4%)
	Bachelor’s degree	33 (13.3%)
	Advanced degree (eg, Masters Doctorates)	37 (14.9%)
	Missing	6 (2.4%)
Employment status^a^	Employed full-time	65 (26.1%)
	Employed part-time	23 (9.2%)
	Unemployed	14 (5.6%)
	Retired	115 (46.2%)
	Disabled	35 (14.1%)
	Student	6 (2.4%)
	Stay at home parent	6 (2.4%)
	Missing	5 (2.0%)
Income	Currently, is your income enough to meet your basic needs for food, housing, clothing, and medical care?	
	Yes	196 (78.7%)
	No	48 (19.3%)
	Missing	5 (2.0%)
Housing	How often in the past 12 months would you say you were worried or stressed about having enough money to pay rent/mortgage?	
	Always	14 (5.6%)
	Usually	16 (6.4%)
	Sometimes	38 (15.3%)
	Rarely	52 (20.9%)
	Never	124 (49.8%)
	Missing	5 (2.0%)
	What is your living situation today?	
	I have a steady place to live	227 (91.2%)
	I have a place to live today, but I am worried about losing it in the future	14 (5.6%)
	I do not have a steady place to live^b^	3 (1.2%)
	Missing	5 (2.0%)
Food insecurity	In the past 12 months, you worried that your food would run out before you got money to buy more	
	Often true	12 (4.8%)
	Sometimes true	37 (14.9%)
	Never true	196 (78.7%)
	Missing	4 (1.6%)
Transportation	In the past 12 months, has lack of reliable transportation kept you from medical appointments, meetings, work, or from getting things needed for daily living?	
	Yes	18 (7.2%)
	No	225 (90.4%)
	Missing	6 (2.4%)

a Employment status=check all that apply. Total *n* > 249.

b Temporarily staying with others, in a hotel, in a shelter, living outside or on the street, on a beach, in a car, abandoned building, bus or train station, or in a park.

### Validating individual EHR data elements

Individually compared with survey responses, the 4 EHR data elements had sensitivity ranging from 81% to 95% and specificity in the range of 65% to 79% (Table 2). As a digital divide marker, the most sensitive (95%) was lack of patient portal use and the most specific was absence of email in the EHR (79%).

**Table 2.** Validation of 4 individual electronic health record data elements as markers of the digital divide: comparing with patient survey self-report (*n*=249).^a^

		Survey self-report	
		Do you have a cell phone?	
		**No**	**Yes**
**EHR cell phone**	**No**	17	56
	**Yes**	4	169
*Survey response missing: 3 (1.2%)*	Sensitivity (95% CI) = 81% (58%-95%)		
	Specificity (95% CI) = 75% (69%-81%)		
	Accuracy (95% CI) = 76% (70%-81%)		
		Do you have an email?	
		**No**	**Yes**
**EHR email**	**No**	45	40
	**Yes**	5	150
*Survey Response Missing: 9 (3.6%)*	Sensitivity (95% CI) = 90% (78%-97%)		
	Specificity (95% CI) = 79% (73%-85%)		
	Accuracy (95% CI) = 81% (76%-86%)		
		Have you been offered access to your online medical records?	
		**No**	**Yes**
**EHR patient portal active**	**No**	51	68
	**Yes**	6	117
*Survey response missing: 7 (2.8%)*	Sensitivity (95% CI) = 89% (79%-96%)		
	Specificity (95% CI) = 65% (58%-72%)		
	Accuracy (95% CI) = 71% (65%-77%)		
		Have you accessed your medical records electronically in the last 12 months?	
		**No**	**Yes**
**EHR patient portal use**	**No**	102	43
	**Yes**	5	96
*Survey response missing: 3 (1.2%)*	Sensitivity (95% CI) = 95% (89%-99%)		
	Specificity (95% CI) = 69% (61%-77%)		
	Accuracy (95% CI) = 80% (75%-85%)		

Absence of electronic health record data element represents a positive marker for the digital divide.

Survey response of “no” represents a positive marker for the digital divide.

a Total *n* varies due to small number (1%-4%) of survey of missing values.

Of note, each survey item was missing for some patients. In sensitivity analyses assessing the impact of missingness on the sensitivity, specificity, and accuracy, results of assigning missing to positive or negative response were contained within the CI of the primary analyses (see Table S3).

### Predicting lack of Internet access with EHR digital divide risk score

**Figure 1.**
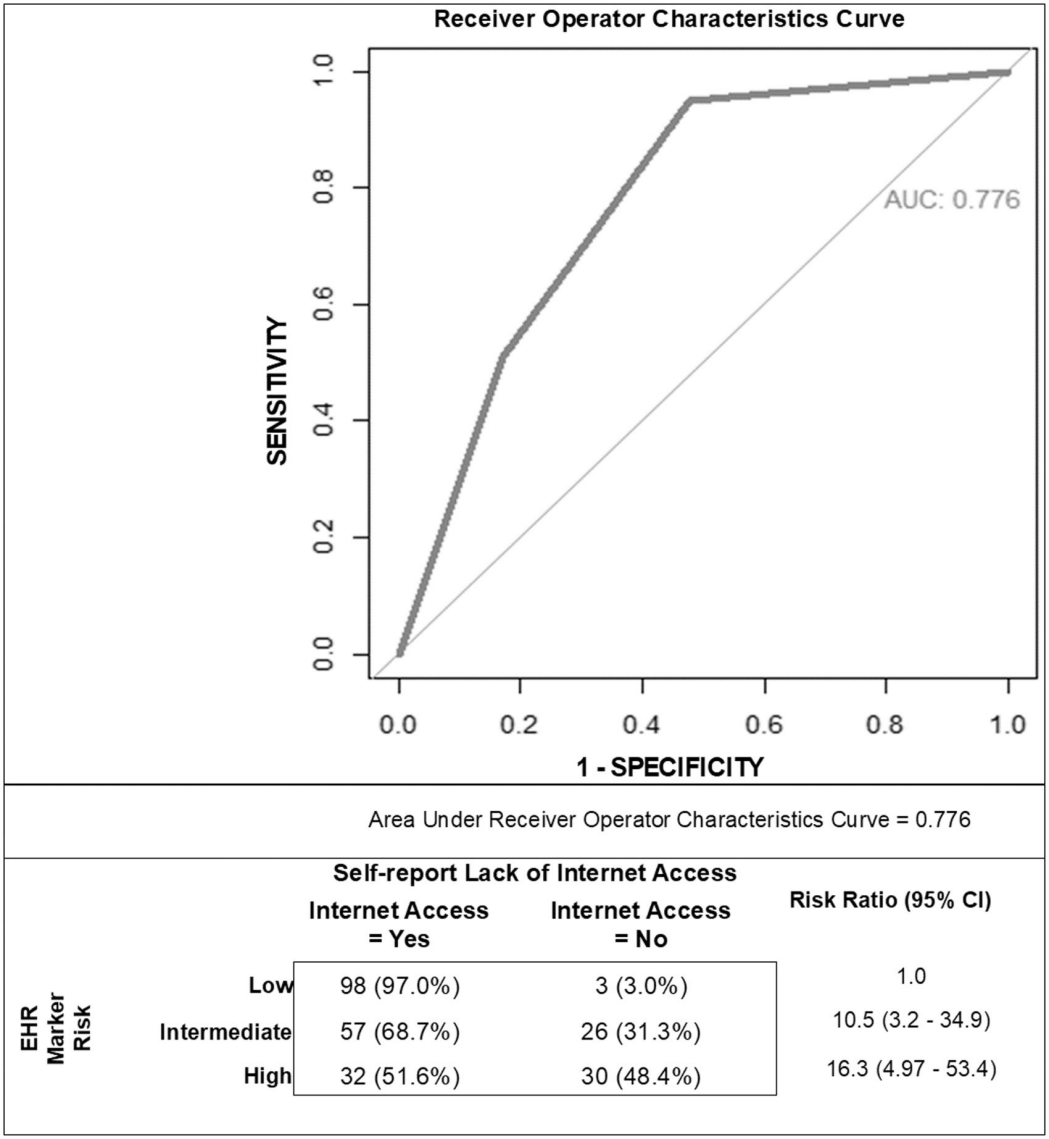
Association of digital divide risk score (high, intermediate, low) of combined 4 digital divide markers and patient self-report of lack of Internet access.

As each of the 4 markers had similar range of sensitivity and specificity, we combined the markers into a risk score with 3 levels (low, intermediate, high). As the EHR digital divide risk increased, self-report of no Internet access increased from 3% to 31.3% to 48.4% (Figure 1) and the ROC curve *c*-statistic was 0.776.

### Association of EHR digital divide risk score with additional digital divide measures

Beyond Internet access, the digital divide can include lower technology use, lower technology use for health, and lower eHealth literacy. In our secondary analyses, we found significant trends in EHR-derived risk for the digital divide and patient self-report on these measures (Table 3). Overall, we found that survey respondents in higher digital divide risk categories (intermediate and high) had lower overall technology use and lower use for health purposes (*P* < .001). Mean digital health literacy scores decreased across for higher levels of digital divide risk (*P* < .001). We also assessed the association of the digital divide risk score with participant characteristics (Table S4).

**Table 3.** Survey domains and risk of digital divide.

Domain	Low risk	Mid risk	High risk	**Trend test** ^a^ ***P***
	(*n*=102)	(*n*=83)	(*n*=64)	
**Goes online to access Internet or send/receive email**				
Yes	97 (95.1%)	58 (69.9%)	32 (50.0%)	<.001
No	3 (2.9%)	22 (26.5%)	30 (46.9%)	
Missing	2 (2.0%)	3 (3.6%)	2 (3.1%)	
**Receives text messages (weekly or daily)**				
Yes	95 (93.1%)	66 (79.5%)	27 (42.2%)	<.001
No	6 (5.9%)	13 (15.7%)	31 (48.4%)	
Missing	1 (1.0%)	4 (4.8%)	6 (9.4%)	
**Sends text messages (weekly or daily)**				
Yes	93 (91.2%)	60 (72.3%)	24 (37.5%)	<.001
No	7 (6.9%)	20 (24.1%)	33 (51.6%)	
Missing	2 (2.0%)	3 (3.6%)	7 (10.9%)	
**Uses cell to access the Internet (weekly or daily)**				
Yes	92 (90.2%)	56 (67.5%)	25 (39.1%)	<.001
No	8 (7.8%)	23 (27.7%)	32 (50.0%)	
Missing	2 (2.0%)	4 (4.8%)	7 (10.9%)	
**Uses technology for health** ^b^				
Yes	97 (95.1%)	45 (54.2%)	26 (40.6%)	<.001
No	4 (3.9%)	34 (41.0%)	36 (56.3%)	
Missing	1 (1.0%)	4 (4.8%)	2 (3.1%)	
**Family/friends help with Internet for health information**				
Yes	22 (21.6%)	18 (21.7%)	10 (15.6%)	.419
No	79 (77.5%)	59 (71.1%)	53 (82.8%)	
Missing	1 (1.0%)	6 (7.2%)	1 (1.6%)	
**eHEALS digital health literacy scale** ^c^				
Mean (SD)	31.4 (6.10)	26.2 (7.60)	22.7 (10.4)	<.001
Median (Min, Max)	31.0 (14.0, 40.0)	28.0 (8.00, 40.0)	26.0 (3.00, 40.0)	
Missing	0 (0%)	4 (4.8%)	7 (10.9%)	

a Cochran Armitage test used for categorical outcomes; Jonckheere-Terpstra test used for quantitative outcomes.

b Do you use any technology such as a computer, cell phone, or the Internet to help you take care of your health and/or manage your health care? For example, some people use technology to search for health information, refill medication, communicate with their doctor, or access medical test results online.

c eHEALS health literacy scale based on cumulative responses to 8 Likert style questions scored 1-5. Higher scores indicate better health literacy.

## Discussion

Among potential data elements identified in the EHR, we identified 4 that were routinely available (cell phone, email, patient portal active, and patient portal used). When each of the 4 EHR data elements was compared with patient self-report, we found high levels of sensitivity, specificity, and overall accuracy. Considering that some time elapsed between the clinical encounter where the EHR data elements were collected and the time that the patient completed the survey, our results represent technology access across 2 timepoints. Considering technology access can change over time, these 2 timepoints present a robust insight into access. Thus, these data elements met criteria for markers of the digital divide. Further, when combined to represent levels of digital divide risk (high, intermediate, lower), the markers predicted overall Internet access (ROC *c*-statistic = 0.77).

Beyond demonstrating the feasibility of using the EHR-based markers as a surrogate for technology access, our results also suggest that the markers are associated with other important aspects of the digital divide. Higher EHR-based digital divide risk was associated with lower self-reported rates of technology use. Importantly, higher EHR-based digital divide risk was also associated with lower digital health literacy, using the validated eHealth instrument. eHealth literacy, known as the ability to seek, find, understand, and appraise health information from electronic sources and apply the knowledge gained to addressing or solving a health problem, has consistently been reported among those at greater risk of the digital divide. This suggests that the individual EHR markers and risk score hold promise when used as a screening mechanism to identify individuals at risk of digital exclusion.

Prior literature suggests particular groups are at risk of inequitable access to digital health tools, including older adults, rural adults, racial and ethnic minorities, those with lower incomes and lower health literacy.^17^ Thus, one of our goals in our recruitment was to assure inclusion of these individuals in our sample, which can provide much greater generalizability to the general population. Although we were successful in including patient groups who have been previously identified as having limited access, we must acknowledge that the sample was recruiting from 1 geographically diverse health care system in the southern United States.

This EHR screening tool may address previously identified EHR clinical decision support implementation challenges,^18^ as it draws on already existing questions/data, not the addition of new questions/screenings. It also does not require training existing or new personnel to implement a new workflow. The ideal implementation approach is to have someone on the medical team (ie, medical assistant, nurse, etc.) ask questions during intake/triage; however, several constraints exist to adding additional questions to busy clinicians workflow and an already burdensome number of EHR screenings, prompts, alerts, etc.^19^^,^^20^ Patients who are at high risk for the digital divide may undergo further screening to identify barriers to digital health, with subsequent referrals to a patient/digital health navigator or for social needs services (ie, if access issues). While our screening approach proposes to use routinely collected EHR data, implementation of digital health supports based on the screening results must consider attitudes toward digital health. Individuals with low socioeconomic status have reported unfavorable attitudes toward digital health.^21–23^ When implementing supports to increase digital health use, health care systems need to carefully consider socioculturally sensitive approaches to ensure successful adoption.^24^

### Limitations

This study has some limitations. Survey responses are inherently subjective, and response bias may exist in those who chose to complete and return the surveys. However, we did have a relatively high response rate for our mail-in survey. We also used home addresses to mail the surveys, thus did not capture responses from individuals who may be experiencing homelessness. Another limitation is that we only sent the survey in English to people who listed English as their preferred language, although the patient portal is available in Spanish. We also only distributed the survey via paper copies, as we felt that would better capture the target population of individuals at risk of the digital divide, though we may have missed responses from individuals who would only respond to electronic versions. The survey predominantly captured the views of individuals aged 55 and above (73.5%), limiting our generalizability to this population. However, as advancing age is associated with greater likelihood of being at risk for the digital divide,^23^ data from this population is particularly useful in the context of this study. As we only incorporated 4 data elements into the risk score, and given the small sample size, our ROC curve had a limited number of cutoff points. While we only used data and patients from 1 health care system, we attempted to enhance generalizability through our purposeful sampling strategy, which assured inclusion of racial and ethnic minorities, older adults, and those from rural areas. Lastly, we chose some criteria from the Digital Health Equity Framework as predictors of the digital divide. However, given the dearth of variables that are known to be associated with digital inequity,^25^ it is possible there are additional variables related to the digital divide than those we examined.

## Conclusions

We identified a risk score for the digital divide using routinely available EHR data elements. The score could be used to identify individuals who may need support when using digital health strategies. After identification, health care systems could provide additional help getting on the patient portal, help conducting telehealth visits, or engaging family members or caregivers with higher digital literacy skills to help patients as they navigate technology and patient provider communication.^26^ eHealth literacy training programs have shown success at increasing patients’ motivation, digital health competencies, and use of digital health tools.^27–29^ Aside from provision of education and social support, this screening tool may provide insight into those lacking technology devices to access digital health. It has been previously suggested that hospitals should screen for device ownership routinely, similar to the Social Determinants of Health screening, and be provided with devices if they are at risk of becoming disconnected.^26^

## Supplementary Material

ooaf004_Supplementary_Data

## Data Availability

The datasets generated during and/or analyzed during the current study are available upon request to the corresponding author.
