# Supplementary material for: Using routinely available electronic health record data elements to develop and validate a digital divide risk score
Source: JAMIA Open. 2025 Feb 4;8(1):ooaf004. doi: 10.1093/jamiaopen/ooaf004 (PMC11792649; doi:10.1093/jamiaopen/ooaf004)
Supplement: ooaf004_Supplementary_Data [file ooaf004_supplementary_data.zip › b3d23_Supplement Table 3.docx]

**Supplement Table 3.** Sensitivity analysis performed by assuming missing values in survey all = Yes or all = No.

|  | Primary Analysis | Sensitivity Analyses | |
| --- | --- | --- | --- |
| Digital Divide Marker | Complete Data from Survey | Assuming Survey Missing = Yes | Assuming Missing Survey = No |
|  |  |  |  |
| Cellular Phone | N = 246 | N = 249 | N = 249 |
| Sensitivity | 81.0% | 81.0% | 79.2% |
| Specificity | 75.1% | 74.6% | 75.1% |
| Accuracy | 75.6% | 75.1% | 75.5% |
|  |  |  |  |
| Email | N = 240 | N = 249 | N = 249 |
| Sensitivity | 90.0% | 90.0% | 88.1% |
| Specificity | 78.9% | 76.4% | 78.9% |
| Accuracy | 81.3% | 79.1% | 81.1% |
|  |  |  |  |
| Portal | N = 242 | N = 249 | N = 249 |
| Sensitivity | 89.5% | 89.5% | 87.5% |
| Specificity | 63.2% | 62.0% | 63.2% |
| Accuracy | 69.4% | 68.3% | 69.5% |
|  |  |  |  |
| Portal Used | N = 246 | N = 249 | N = 249 |
| Sensitivity | 95.3% | 95.3% | 94.5% |
| Specificity | 69.1% | 68.3% | 69.1% |
| Accuracy | 80.5% | 79.9% | 80.3% |
