# Supplementary material for: Using routinely available electronic health record data elements to develop and validate a digital divide risk score
Source: JAMIA Open. 2025 Feb 4;8(1):ooaf004. doi: 10.1093/jamiaopen/ooaf004 (PMC11792649; doi:10.1093/jamiaopen/ooaf004)
Supplement: ooaf004_Supplementary_Data [file ooaf004_supplementary_data.zip › 8d65a_Supplement Table 4.docx]

**Supplement Table 4.** Association of Participant Characteristics and Digital Divide Risk Score level. P-values from chi squared test or fishers exact test when counts were less than 5.

|  | **Low Risk** | **Mid Risk** | **High Risk** | **P-value** |
| --- | --- | --- | --- | --- |
|  | **(N=102)** | **(N=83)** | **(N=64)** |  |
| **Age** |  |  |  |  |
| 18-34 | 16 (15.7%) | 10 (12.0%) | 6 (9.4%) | 0.751 |
| 35-54 | 15 (14.7%) | 13 (15.7%) | 6 (9.4%) |  |
| 55-64 | 18 (17.6%) | 17 (20.5%) | 15 (23.4%) |  |
| 65+ | 53 (52.0%) | 43 (51.8%) | 37 (57.8%) |  |
| **Sex** |  |  |  |  |
| Female | 56 (54.9%) | 40 (48.2%) | 32 (50.0%) | 0.64 |
| Male | 46 (45.1%) | 43 (51.8%) | 32 (50.0%) |  |
| **Race** |  |  |  |  |
| Black/African American | 11 (10.8%) | 36 (43.4%) | 11 (17.2%) | <0.001 |
| Others | 21 (20.6%) | 35 (42.2%) | 11 (17.2%) |  |
| White, Non-Hispanic | 70 (68.6%) | 12 (14.5%) | 42 (65.6%) |  |
| **Ethnicity** |  |  |  |  |
| Hispanic/Latino | 8 (7.8%) | 14 (16.9%) | 4 (6.3%) | 0.00189 |
| Not Hispanic/Latino | 93 (91.2%) | 60 (72.3%) | 58 (90.6%) |  |
| Patient Refused | 1 (1.0%) | 9 (10.8%) | 2 (3.1%) |  |
| **Location** |  |  |  |  |
| Metropolitan or Micropolitan | 48 (47.1%) | 29 (34.9%) | 34 (53.1%) | 0.224 |
| Rural | 25 (24.5%) | 22 (26.5%) | 14 (21.9%) |  |
| Small Town | 29 (28.4%) | 32 (38.6%) | 16 (25.0%) |  |
| **Insurance** |  |  |  |  |
| Commercial | 48 (47.1%) | 40 (48.2%) | 28 (43.8%) | 0.974 |
| Medicaid | 9 (8.8%) | 6 (7.2%) | 5 (7.8%) |  |
| Medicare | 45 (44.1%) | 37 (44.6%) | 31 (48.4%) |  |
| **Education Status** |  |  |  |  |
| Did not graduate high school | 2 (1.9%) | 11 (13.3%) | 15 (23.4%) | <0.001 |
| Finished high school or GED | 20 (19.6%) | 35 (42.2%) | 22 (34.4%) |  |
| Some College | 19 (18.6%) | 21 (25.3%) | 7 (10.9%) |  |
| Associate's Degree | 16 (15.7%) | 2 (2.4%) | 3 (4.7%) |  |
| Bachelor's Degree | 18 (17.6%) | 7 (8.4%) | 8 (12.5%) |  |
| Advanced College Degree (e.g., Masters, Doctorates) | 26 (25.5%) | 5 (6.0%) | 6 (9.4%) |  |
| Missing | 1 (0.9%) | 2 (2.4%) | 3 (4.7%) |  |
| **Employment Status** |  |  |  |  |
| Employed full-time | 30 (29.4%) | 23 (27.7%) | 12 (18.8%) | 0.289 |
| Employed part-time | 8 (7.8%) | 10 (12.0%) | 5 (7.8%) | 0.556 |
| Retired | 47 (46.1%) | 36 (43.4%) | 32 (50.0%) | 0.726 |
| Disabled | 12 (11.8%) | 10 (12.0%) | 13 (20.3%) | 0.247 |
| Unemployed | 3 (2.9%) | 6 (7.2%) | 5 (7.8%) | 0.319 |
| Student | 3 (2.9%) | 1 (1.2%) | 2 (3.1%) | 0.669 |
| Stay at home parent | 4 (3.9%) | 1 (1.2%) | 1 (1.6%) | 0.577 |
| Missing | 1 (1.0%) | 3 (3.6%) | 1 (1.6%) |  |
| **Currently, is your income enough to meet your basic needs for food, housing, clothing, and medical care?** |  |  |  |  |
| Yes | 85 (83.3%) | 60 (72.3%) | 51 (79.7%) | 0.201 |
| No | 14 (13.7%) | 21 (25.3%) | 13 (20.3%) |  |
| Missing | 3 (2.9%) | 2 (2.4%) | 0 (0%) |  |
| **How often in the past 12 months would you say you were worried or stressed about having enough money to pay rent/mortgage?** |  |  |  |  |
| Always | 6 (5.9%) | 4 (4.8%) | 4 (6.3%) | 0.869 |
| Usually | 6 (5.9%) | 5 (6.0%) | 5 (7.8%) |  |
| Sometimes | 15 (14.7%) | 12 (14.5%) | 11 (17.2%) |  |
| Rarely | 55 (53.9%) | 37 (44.6%) | 32 (50.0%) |  |
| Never | 6 (5.9%) | 4 (4.8%) | 4 (6.3%) |  |
| Missing | 2 (2.0%) | 3 (3.6%) | 0 (0%) |  |
| **What is your living situation today?** |  |  |  |  |
| I have a steady place to live | 95 (93.1%) | 74 (89.2%) | 58 (90.6%) | 0.721 |
| I have a place to live today, but I am worried about losing it in the future | 4 (3.9%) | 5 (6.0%) | 5 (7.8%) |  |
| I do not have a steady place to live ^2^ | 1 (1.0%) | 1 (1.2%) | 1 (1.6%) |  |
| Missing | (2.0%) | 3 (3.6%) | 0 (0%) |  |
| **In the past 12 months, you worried that your food would run out before you got money to buy more.** |  |  |  |  |
| Often true | 5 (4.9%) | 5 (6.0%) | 2 (3.1%) | 0.032 |
| Sometimes true | 7 (6.9%) | 19 (22.9%) | 11 (17.2%) |  |
| Never true | 88 (86.3%) | 57 (68.7%) | 51 (79.7%) |  |
| Missing | 2 (2.0%) | 2 (2.4%) | 0 (0%) |  |
| **In the past 12 months, has lack of reliable transportation kept you from medical appointments, meetings, work, or from getting things needed for daily living?** |  |  |  |  |
| Yes | 5 (4.9%) | 7 (8.4%) | 6 (9.4% | 0.689 |
| No | 95 (93.1%) | 73 (88.0%) | 57 (89.1%) |  |
| Missing | 2 (2.0%) | 3 (3.6%) | 1 (1.6%) |  |
