# Supplementary material for: Using routinely available electronic health record data elements to develop and validate a digital divide risk score
Source: JAMIA Open. 2025 Feb 4;8(1):ooaf004. doi: 10.1093/jamiaopen/ooaf004 (PMC11792649; doi:10.1093/jamiaopen/ooaf004)
Supplement: ooaf004_Supplementary_Data [file ooaf004_supplementary_data.zip › 9d2cf_Supplement 1.docx]

Supplement File 1. Survey mailed to participants.

| **Questions 1 - 9 ask about your use of technology** | |
| --- | --- |
| **1.** | **Do you have access to the Internet in your home?** |
| - Yes - No - Unsure | |
| **2.** | **Do you ever go on-line to access the Internet (i.e., “World Wide Web”) or to send and receive e-mail?** |
| - Yes - No | |
| **3.** | **When you use the Internet, do you access it through... (please mark all that apply)** |
|  | - A regular dial-up telephone line - Broadband such as DSL, cable, or FiOS - A cellular network (i.e., phone, tablet, 3G/4G/5G/LTE) - A wireless network (Wi-Fi) - Other (please specify): ________________________________________ - I do not use the internet. |
| **4.** | **Do you use a cellular phone (i.e., “cell phone” or mobile phone)?** |
| - Yes - No | |
| **5.** | **Do you have your cell phone number on file with your health care provider(s)?** |
| - Yes - No - Unsure - I do not use a cell phone | |
| **6.** | **Do you want to receive text messages from your health care provider(s)?** |
| - Yes - No - I already receive text messages from my health care provider(s) - I do not use a cell phone | |
| **7.** | **If you answered “No” to #6, why don’t you want to receive text messages from your health care provider(s)? (Mark all that apply)** |
| - Cost - Not convenient - Invasion of my privacy - I don’t trust text messages - Other (please specify): ________________________________________ - Not Applicable - I do want to receive text messages from my health care provider(s) - I do not use a cell phone (please skip to question 9) | |
| **8.** | **How often do you do the following:** |
| \|  \| Many times a day \| A few times a day \| A few times a week \| Rarely \| Never \| \| --- \| --- \| --- \| --- \| --- \| --- \| \| a. How often do you receive text messages on your cell phone? \| □ \| □ \| □ \| □ \| □ \| \| b. How often do you send text messages on your cell phone? \| □ \| □ \| □ \| □ \| □ \| \| c. How often do you access the internet on your cell phone? \| □ \| □ \| □ \| □ \| □ \|     **Please Continue to the Next Page** | |
|  | |
| **9.** | **Do you have an email address?** |
| - Yes - No - Unsure | |
| **Questions 10 – 12 ask you some questions about your medical records.**  ***Medical records are defined as medical history, such as laboratory test results, clinical notes, and current list of medications.*** | |
| **10.** | **Have you ever been offered online access to your medical records by your health care provider?** |
| - Yes - No | |
| **11.** | **How many times did you access your online medical record in the last 12 months?** |
| - 0 - 1 to 2 times - 6 to 9 times - 10 times or more | |
| **12.** | **In the past 12 months, have you used your online medical record to…** |
| \|  \| Yes \| No \| \| --- \| --- \| --- \| \| a. Look up test results? \| □ \| □ \| \| b. Securely message your health care provider or staff (for example, e-mail)? \| □ \| □ \| \| c. Download your health information to your computer or mobile device, such as a cellphone or tablet? \| □ \| □ \| | |
| **Questions 13 - 19 ask about your use of the Internet and technology in managing your healthcare.** | |
| **13.** | [**Do you use any technology such as a computer, cell phone, or the Internet to help you take care of your health and/or manage your health care? For example, some people use technology to search for health information, refill medications, communicate with their doctor, or access medical test results online.**](http://dx.doi.org/10.1007/s11606-015-3222-9) |
| - Yes - No | |
| **14.** | **Which of the following have you used to help you take care of your health and/or manage your health care? (Check all that apply)** |
| - Computers - Internet - Email - Text messaging - Social media (i.e., Facebook) - Video calling (i.e., Skype, Zoom, Facetime) - Mobile apps (i.e., cell phone programs) - I do not use technology to help take care of or manage my health care | |
| **Please Continue to the Next Page** | |
|  | |
| **15.** | **H**[**ow frequently do you use technology to help you take care of your health and/or manage health care?**](http://dx.doi.org/10.1007/s11606-015-3222-9) |
| - Less than once per month - 1-3 times per month - 1-5 times per week - Daily - I do not use technology for my health | |
| **16.** | [**Which of the following activities have you performed using the computer, the Internet, or a cell phone? (Check all that apply)**](http://dx.doi.org/10.1007/s11606-015-3222-9)**:** |
| - - Searched for health information   - Bought medications or medical supplies - Communicated with my health care provider (using the internet or a mobile device application) - Used the Internet to help me make treatment decisions - Visited an online support group or social networking website regarding health issues - Used the computer to help me track my medication list - Used the computer to help me keep track of my medical information (such as test results, blood pressure, or sugar testing results) - Used a mobile phone app for a health problem - Participated in a health-related competition or game online - Other (please specify): _____________________________________________ | |
| **17.** | [**The following questions ask about your use of the Internet for health information**](http://dx.doi.org/10.1007/s11606-015-3222-9)**:** |
| \|  \| Strongly Agree \| Agree \| Undecided \| Disagree \| Strongly Disagree \| \| --- \| --- \| --- \| --- \| --- \| --- \| \| 1. I know *what* health resources are available on the Internet. \| □ \| □ \| □ \| □ \| □ \| \| 1. I know *where* to find helpful health resources on the Internet. \| □ \| □ \| □ \| □ \| □ \| \| 1. I know *how to find* helpful health resources on the Internet. \| □ \| □ \| □ \| □ \| □ \| \| 1. I know *how to use the Internet* to answer my questions about health \| □ \| □ \| □ \| □ \| □ \| \| 1. I know *how to use the health information* I find on the Internet. \| □ \| □ \| □ \| □ \| □ \| \| 1. I have the *skills* I need to *evaluate* the health resources I find on the Internet. \| □ \| □ \| □ \| □ \| □ \| \| 1. I can tell *high-quality* from *low-quality* health resources on the Internet. \| □ \| □ \| □ \| □ \| □ \| \| 1. I feel *confident* using information from the Internet to make health decisions. \| □ \| □ \| □ \| □ \| □ \| | |
| **18.** | **Thinking about the LAST time you went online for health or medical information; did you go online to look for information related to your own health or medical situation or someone else's health or medical situation?** |
| - Your own health or medical situation - Someone else’s health or medical situation - Both, your own and someone else’s health or medical situation - I have never gone online for health or medical information   **Please Continue to the Next Page** | |
|  | |
| **19.** | **In the past 3 months, have you had someone else help you look up information about your health online or through a mobile application?** |
| - Yes - No | |
| **Questions 20 – 26 are about you.** | |
| **20.** | **What is the highest grade of school that you have completed?** |
| - Eighth grade or less - Some high school - Finished high school or GED - Some college - Associate’s Degree - Bachelor’s Degree - Advanced College Degree (e.g., Masters, Doctorates) | |
| **21.** | **What is your current employment status? (Please check all that apply)** |
| - Employed, full-time - Employed, part-time - Retired - Disabled - Unemployed - Student - Stay at home parent | |
| **22.** | **How often in the past 12 months would you say you were worried or stressed about having enough money to pay your rent/mortgage? Would you say:** |
| - Always - Usually - Sometimes - Rarely - Never | |
| **23.** | **Currently, is your income enough to meet your basic needs for food, housing, clothing, and medical care?** |
| - Yes - No | |
| **24.** | **What is your living situation today?** |
| - I have a steady place to live - I have a place to live today, but I am worried about losing it in the future - I do not have a steady place to live (temporarily staying with others, in a hotel, in a shelter, living outside on the street, on a beach, in a car, abandoned building, bus or train station, or in a park) | |
| **25.** | **In the past 12 months, you worried that your food would run out before you got money to buy more.** |
| - Often True - Sometimes True - Never True | |
| **26.** | **In the past 12 months, has lack of reliable transportation kept you from medical appointments, meetings, work or from getting things needed for daily living?** |
| - Yes - No   **Please Continue to the Next Page** | |
| **Lastly, questions 27 – 31 ask about your healthcare experience.** | |
| **27.** | **Medical doctors provide patients with diagnoses of disease and/or treatment recommendations to promote, maintain or restore a patient’s health. In general, would you say your view of medical doctors is…** |
| - Mostly Positive - Neither Positive nor Negative - Mostly Negative | |
| **28.** | **Overall, how would you rate the quality of health care you received in the past 12 months?** |
| - Excellent - Very Good - Good - Fair - Poor | |
| **29.** | **In the past 12 months, when getting care for a medical problem, was there a time when you had to wait for test results longer than you thought reasonable?** |
| - Yes - No - Unsure | |
| **30.** | **In the past 12 months…** |
| \|  \| All of the time \| Most of the time \| Some of the time \| A little of the time \| None of the time \| \| --- \| --- \| --- \| --- \| --- \| --- \| \| 1. How often do you have someone (like a family member, friend, hospital/ clinic worker or caregiver) help you read hospital materials? \| □ \| □ \| □ \| □ \| □ \| \| 1. How often do you have problems learning about your medical condition because of difficulty understanding written information? \| □ \| □ \| □ \| □ \| □ \| \| 1. How often do you feel confident filling out forms by yourself? \| □ \| □ \| □ \| □ \| □ \| \| 1. How often did doctors, nurses, or other health professionals explain things in a way you could understand? \| □ \| □ \| □ \| □ \| □ \| \| 1. How often did doctors, nurses, or other health professionals involve you in a decision about your health care as much as you wanted? \| □ \| □ \| □ \| □ \| □ \| \| 1. How often did doctors, nurses, or other health professionals give you the chance to ask all of the health-related questions you had? \| □ \| □ \| □ \| □ \| □ \|     **Please Continue to the Next Page** | |
| **31.** | [**The following is a list of common medical issues**](http://onlinelibrary.wiley.com/doi/10.1002/art.10993/abstract)**.**  *Please indicate if you currently have the problem in the first column. If you do have the problem, in the second column, please indicate if you receive medications or some other type of treatment for the problem. In the third column, please also indicate if the problem limits any of your activities.* |
| \|  \| Do you have the problem? \| \| Do you receive treatment for it? \| \| Does it limit your activities? \| \| \| --- \| --- \| --- \| --- \| --- \| --- \| --- \| \| *Problem* \| *Yes* \| *No* \| *Yes* \| *No* \| *Yes* \| *No* \| \| 1. Heart Disease \| ☐ \| ☐ \| ☐ \| ☐ \| ☐ \| ☐ \| \| 1. High Blood Pressure \| ☐ \| ☐ \| ☐ \| ☐ \| ☐ \| ☐ \| \| 1. Lung Disease \| ☐ \| ☐ \| ☐ \| ☐ \| ☐ \| ☐ \| \| 1. Diabetes \| ☐ \| ☐ \| ☐ \| ☐ \| ☐ \| ☐ \| \| 1. Ulcer or stomach disease \| ☐ \| ☐ \| ☐ \| ☐ \| ☐ \| ☐ \| \| 1. Kidney disease \| ☐ \| ☐ \| ☐ \| ☐ \| ☐ \| ☐ \| \| 1. Liver disease \| ☐ \| ☐ \| ☐ \| ☐ \| ☐ \| ☐ \| \| 1. Anemia or other blood disease \| ☐ \| ☐ \| ☐ \| ☐ \| ☐ \| ☐ \| \| 1. Cancer (Please specify type(s))  _______________________________   _______________________________ \| ☐  ☐ \| ☐  ☐ \| ☐  ☐ \| ☐  ☐ \| ☐  ☐ \| ☐  ☐ \| \| 1. Depression \| ☐ \| ☐ \| ☐ \| ☐ \| ☐ \| ☐ \| \| 1. Osteoarthritis, degenerative arthritis \| ☐ \| ☐ \| ☐ \| ☐ \| ☐ \| ☐ \| \| 1. Back pain \| ☐ \| ☐ \| ☐ \| ☐ \| ☐ \| ☐ \| \| 1. Rheumatoid Arthritis \| ☐ \| ☐ \| ☐ \| ☐ \| ☐ \| ☐ \| \| 1. Other (please specify)  _______________________________ \| ☐ \| ☐ \| ☐ \| ☐ \| ☐ \| ☐ \|     **END OF SURVEY** | |
|  | |
