# Supplementary material for: Using routinely available electronic health record data elements to develop and validate a digital divide risk score
Source: JAMIA Open. 2025 Feb 4;8(1):ooaf004. doi: 10.1093/jamiaopen/ooaf004 (PMC11792649; doi:10.1093/jamiaopen/ooaf004)
Supplement: ooaf004_Supplementary_Data [file ooaf004_supplementary_data.zip › 1b6cb_Supplement Table 2.docx]

**Supplement Table 2.** Comparing Respondents and non-respondents for Digital Divide Survey.

|  |  | **Respondents** | **Non-respondents** | **Chi-Squared P-Value** |
| --- | --- | --- | --- | --- |
|  | **Variable** | **N=249** | **(N=383)** |  |
|  | Age |  |  | 0.0216 |
|  | 18-34 | 32 (12.9%) | 63 (16.4%) |  |
| Demographic Characteristics | 35-54 | 34 (13.7%) | 83 (21.7%) |  |
|  | 55-64 | 50 (20.1%) | 62 (16.2%) |  |
|  | 65+ | 133 (53.4%) | 175 (45.7%) |  |
|  | Gender |  |  | 1 |
|  | Female | 128 (51.4%) | 198 (51.7%) |  |
|  | Male | 121 (48.6%) | 185 (48.3%) |  |
|  | Race |  |  | <0.001 |
|  | White | 124 (49.8%) | 116 (30.3%) |  |
|  | Black | 58 (23.3%) | 105 (27.4%) |  |
|  | Other Race | 67 (26.9%) | 162 (42.3%) |  |
|  | Ethnicity |  |  | <0.001 |
|  | Hispanic/Latino | 26 (10.4%) | 87 (22.7%) |  |
|  | Not Hispanic/Latino  Missing | 211 (84.7%)  12 (4.8%) | 274 (71.5%)  22 (5.7%) |  |
| Urban-Rural location | Metropolitan or Micropolitan | 111 (44.6%) | 174 (45.4%) | 0.957 |
|  | Small Town | 77 (30.9%) | 119 (31.1%) |  |
|  | Rural | 61 (24.5%) | 90 (23.5%) |  |
| Health Insurance | Commercial | 116 (46.6%) | 189 (49.3%) | 0.0651 |
|  | Medicaid | 20 (8.0%) | 145 (37.9%) |  |
|  | Medicare | 113 (45.4%) | 49 (12.8%) |  |
